# Large-scale network topography of stroke predicts functional outcome after mechanical thrombectomy

**DOI:** 10.1093/braincomms/fcaf285

**Published:** 2025-08-28

**Authors:** Antonio Luigi Bisogno, Lorenzo Pini, Sofia Raccanello, Giorgia Adamo, Joseph Domenico Gabrieli, Alessandro Salvalaggio, Anna Maria Basile, Claudio Baracchini, Maurizio Corbetta

**Affiliations:** Department of Neuroscience, Università di Padova, Padova 35128, Veneto, Italy; Department of Neuroscience, Università di Padova, Padova 35128, Veneto, Italy; Department of Neuroscience, Università di Padova, Padova 35128, Veneto, Italy; Department of Neuroscience, Università di Padova, Padova 35128, Veneto, Italy; Department of Neuroscience, Università di Padova, Padova 35128, Veneto, Italy; Department of Neuroscience, Università di Padova, Padova 35128, Veneto, Italy; Azienda Ospedaliera Università di Padova, Padova 35128, Veneto, Italy; Department of Neuroscience, Università di Padova, Padova 35128, Veneto, Italy; Azienda Ospedaliera Università di Padova, Padova 35128, Veneto, Italy; Department of Neuroscience, Università di Padova, Padova 35128, Veneto, Italy; Azienda Ospedaliera Università di Padova, Padova 35128, Veneto, Italy; Veneto Institute of Molecular Medicine (VIMM), Padova 35129, Veneto, Italy

**Keywords:** stroke, recovery, thrombectomy, neural networks

## Abstract

Mechanical thrombectomy effectively restores blood flow in patients with acute ischaemic stroke caused by large vessel occlusion. While mechanical thrombectomy has improved functional outcomes, 35%–60% of patients still experience residual disabilities. Typically, patients are selected for mechanical thrombectomy based on degree of hypoperfusion around the core measured on a vascular atlas. This study had two aims: (i) to evaluate the prognostic value of lesion topography onto functional outcome at 3 months post-mechanical thrombectomy, when the lesion is localized either onto a vascular atlas or large-scale, functional or structural, network atlases; and (2) to examine patterns of post-stroke structural and functional disconnection significantly related to the most common stroke functional outcome scale, i.e. the modified Rankin scale at 3 months post-event. A retrospective analysis was conducted on 70 acute stroke patients who underwent mechanical thrombectomy at the Padua University Hospital (January 2018–June 2022). Inclusion criteria involved first ever ischaemic strokes with anterior circulation large vessel occlusion. Imaging data from sub-acute structural MRI and CT scans were used to estimate indirect structural and functional disconnections. Outcome measures included the modified Rankin Scale at 3 months, with prediction analysis performed using Lasso regression across vascular, grey matter and white matter atlases. Three-month modified Rankin Scale was best predicted using Yeo's functional atlas (*R*^2^ = 0.382), followed by the functional white matter atlas (*R*^2^ = 0.338); the vascular atlas yielded the weakest prediction (*R*^2^ = 0.146). Lesion damage to the corticospinal tract and corona radiata was significantly associated with the modified Rankin Scale. Functional disconnection significantly correlated with disability, particularly in sensorimotor, dorsal attention (DAN) and visual networks. Structural disconnections in the corticospinal tract, corpus callosum, corona radiata, thalamic radiation and left inferior and superior longitudinal fasciculus were also associated with poor functional outcome. This study demonstrates that lesion topography embedded in a network framework provides a more robust prediction of functional outcome. These findings emphasize the importance of understanding network alterations to enhance recovery prediction and optimize treatment strategies for stroke patients. Further research should explore the integration of network-based assessments in clinical practice for evaluating revascularization treatment eligibility.

## Teaser

Bisogno *et al*. report that considering stroke lesions in a large-scale network atlas in comparison to a vascular atlas, typically used in clinical practice, is more informative on functional outcome and disability of patients. These results provide pathophysiological implications on recovery mechanisms following stroke.

## Graphical Abstract

**Graphical Abstract:**
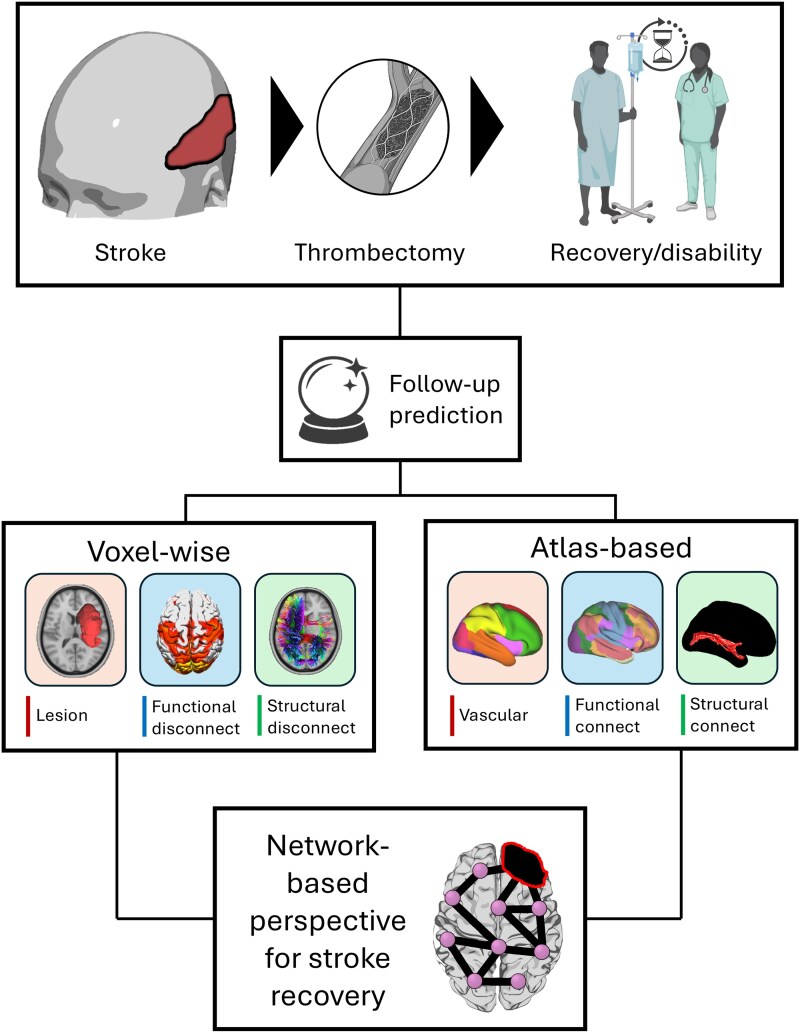

## Introduction

Mechanical thrombectomy (MT) restores blood flow after the acute occlusion of a large brain vessel causing ischaemic stroke.^1^ MT prevents damage to the ‘penumbra’ brain region suffering from reversible ischemia. The application of this treatment in selected patients, as demonstrated by several randomized control trials, has led to significant improvement of functional outcome.^2-5^ However, only approximately 40–75% of patients have a good outcome, while the remaining 35–60% experience residual disabilities.^6,7^ This heterogeneity of ‘treatment effects’ is partially dependent on well-established pre-treatment and post-treatment prognostic factors.^4,5^ Pre-treatment prognostic features are essential to identify those patients who may benefit from MT, thus moving towards patient-tailored medicine. These include, among others, clinical parameters [e.g. age, pre-stroke modified Rankin Scale (mRS), National Institutes Health Stroke Scale (NIHSS) at presentation, time between event and revascularization] and imaging parameters such as the Alberta Stroke Programme Early CT score (ASPECTS), ischaemic core and penumbra volumes assessed by magnetic resonance imaging (MRI) or computed tomography perfusion (CTP) imaging protocols.^8-10^ Ischaemic core/penumbra volumes are parameters that identify patients who might benefit from thrombectomy even in a ‘late window’ setting, thus significantly widening the eligible population for treatment.^11,12^ ASPECTS is a semi-quantitative measure (1 point is subtracted from 10 for any evidence of early ischaemic damage for each of the defined regions) providing coarse topographical data of early CT alterations following middle cerebral artery occlusions according to a cerebrovascular distribution.^10^ Post-treatment prognostic factors include numerous additional clinical, radiological and neuro-sonological variables [e.g. expanded Thrombolysis in Cerebral Infarction score (eTICI) score, NIHSS at 24 h, final infarct volume, haemorrhagic transformation].^6,13-16^

While these features have helped define personalized treatment for stroke patients, a large body of literature has progressively emphasized the role of lesion location on severity and potential for neuroplastic recovery.^17-19^ Weaver *et al*. showed that right parietal lobe, left frontal and temporal lobes and left thalamus were most predictive for the development of post-stroke cognitive impairment.^20^ Rosso and Samson observed that the perfusion–diffusion-weighted imaging volumetric mismatch poorly predicted outcome, whereas lesion location provided a better prediction.^21^ Recently, a retrospective analysis on 162 stroke patients with large vessel occlusion (LVO) who underwent MT demonstrated that the preservation of the corticospinal tract (i.e. unaffected by the ischaemic core based on acute CTP) was associated with a reduced risk of a worse functional outcome.^22^

In addition to direct anatomical damage, remote structural and functional changes following focal lesions correlate with deficits in different behavioural domains.^23^ When considering extensive large-scale network disruption, the weakening of inter-hemispheric functional connectivity and the intensification of intra-hemispheric segregation provide a better explanation of acute impairment.^24-27^ The prediction of acute stroke severity was also significantly improved using indirect structural and functional disconnection measures derived from embedding the lesion location into normative connectomes.^28^ This operational framework has been developed to estimate brain disconnections without the need of patient data relying on large normative datasets of connectivity. With this approach,^29^ lesion segmentation from clinical scans is used as a seed to estimate connections in the normative dataset. The resulting maps are to be considered a rough estimation of which regions are likely disconnected in a specific patient.

Finally, the role of large-scale networks has also been extensively related to potential recovery following stroke when considering the concept of structural reserve and bimodal balance–recovery following a focal lesion.^30^ Specifically, structural reserve can be defined as the quantity of strategic neural pathways and relays that are spared by the lesion and can reallocate previous or outsource new functions, as reported in an extensive review by Di Pino *et al*.^31^

This emerging body of literature emphasizes the predictive value of lesion topography and calls for an efficient integration into clinical practice, where volumetric and vascular data currently guide the neurological evaluation and revascularization protocols entirely.^4^ This study had two main objectives to assess this issue. Firstly, we evaluated the prognostic value of lesion topography on functional outcome and disability 3 months after MT, localizing the lesion onto a vascular atlas or large-scale, functional or structural, network atlases. If prognosis depends on the vascular distribution of the lesion, a vascular atlas shall provide the best prediction of outcome. However, if lesions impair structural and functional networks, then a prediction based on structural or functional connectivity shall provide higher prediction. This issue is very relevant since current eligibility criteria for MT rely on volumetric and vascular scores (i.e. ASPECTS, vascular hypoperfusion ratio). Secondly, we took advantage of the previously described indirect disconnectivity approach to examine patterns of post-stroke structural and functional disconnection significantly related to the most common stroke disability scale, i.e. the mRS. We retrospectively analysed sub-acute imaging and behavioural data of stroke patients with acute LVO in the anterior circulation who underwent MT. We performed all analysis considering lesions derived from routinely performed scans (i.e. sub-acute structural MRI/CT scans) and measures of outcome (i.e. the mRS at 3 months) to emphasize potential clinical applicability and future translation to a pre-treatment setting.

## Materials and methods

### Participants

The study population included acute stroke patients admitted to the Stroke Unit of the Padua University Hospital who underwent MT from January 2018 to June 2022. The inclusion criteria were as follows: (i) first ever ischaemic stroke, (ii) LVO eligible for MT according to current international guidelines, and (iii) anterior circulation stroke. Exclusion criteria were as follows: (i) previous stroke based on clinical imaging, (ii) no LVO, (iii) patients ineligible for MT, (iv) non-available advanced neuroimaging (i.e. perfusion Computer Tomography (pCT) or perfusion weighted imaging (PWI)), (v) patients undergoing thrombolysis, and (vi) lack of follow-up clinical data.

On admission, all patients were clinically evaluated by the NIHSS. Pre-event mRS data were also collected. Before performing MT, all patients underwent CT scans and advanced CTP imaging according to International Guidelines on revascularization therapy. We performed CT and MRI examinations following MT. During hospitalization and at discharge, patients were re-evaluated by the NIHSS and the mRS; final disability was assessed at 3 months by the mRS.

To thoroughly characterize the clinical sample, a complete dataset was collected for each patient: demographics (gender, age), vascular risk factors (such as arterial hypertension, atrial fibrillation, current/past cigarette smoking, diabetes, hypercholesterolaemia, hyperlipidaemia, history of transient ischemic attack/acute ischemic stroke, heart failure, coronary artery disease), severity of neurovascular syndrome (NIHSS at baseline, at discharge), neuroradiological features (baseline ASPECTS, occluded vessel at angio-CT, ischaemic volume at CTP), timing (onset-to-door, door-to-groin, groin-to-recanalization), degree of recanalization (the eTICI),^13^ procedural complications (haemorrhagic transformation categorized by the Heidelberg bleeding classification^32^ and systemic complications (pneumonia, respiratory distress, cardiac failure, renal failure, etc.).

### Imaging data and lesion maps

Sub-acute lesions following MT were obtained from fluid attenuation inversion recovery (FLAIR) and CT scans (on average on 7 ± 3.5 days). FLAIR images were preferred when both were present due to higher resolution. Perfusion imaging data were also collected but will not be considered for the following analysis.

Patient's sub-acute lesions were manually segmented and verified by a neurology consultant (A.L.B.) using ITK-SNAP tool software. Lesions were normalized with a non-linear transformation using the Advanced Normalization tool onto the MNI brain atlas,^33^ with a cost function mask approach. The normalization matrix was then applied to the lesion mask through a nearest neighbour interpolation function.

### Structural and functional disconnection analysis

We used the BCB toolkit^34^ to estimate indirectly the structural disconnection caused by a lesion. The inference about the affected structural pathways is made by embedding the lesion into a normative structural connectome obtained from a sample of healthy subjects. The lesions were normalized to MNI space resampled to 1 × 1 × 1 mm, to match the space of the tracts included in the BCB toolkit. For each voxel, the likelihood that a white matter bundle directly connected with the lesion passes through it is calculated.

Functional disconnection was computed according to previous studies.^35,36^ Specifically, normalized lesions were resampled to 2 mm isotropic, binarized and used as seed region of interest for functional connectivity computation. Each lesion was embedded into a normative connectome of 173 subjects from the Human Connectome Project (HCP) dataset scanned with a 7T MRI machine. We used the same procedure described in our previous study.^37^ Briefly, a principal component analysis of the within-lesion connectivity matrix was applied to identify the main axis of variance, which was used to reshape the original lesion. The reshaped lesion was used as seed region to compute the functional connectivity profile between the lesion and the rest of the voxels of the brain, resulting in a whole brain disconnectivity map. It is worth noting that this procedure parallels structural disconnection maps as an indirect measure of brain ‘disconnectivity’ between the lesion and the brain.

### Normative atlases

Three different atlases were used to investigate the prediction of the lesion on functional outcome (i.e. mRS at 3 months). The vascular atlas^38^ represents arterial territories in a 3D space based on stroke lesion distributions in 1298 acute patients. The atlas encloses supra- and infra-tentorial regions created by a mixture of anatomical and vascular criteria. Specifically, this atlas defines four major supra- and infra-tentorial arterial territories: vertebro-basilar, anterior, middle and posterior Cerebral Arteries, and sub-territories (cerebellar arterial territories, basilar, thalamoperforating and lenticulostriate), distributed in two hierarchical levels. Level 2 represents the major vascular territories (anterior cerebral artery [ACA], middle cerebral artery [MCA], posterior cerebral artery [PCA] and vertebro-basilar [VB]), while Level 1 includes 32 subdivisions. We utilized the latter since this atlas provides better anatomical resolution of vascular territories, including subdivisions for the middle cerebral artery (commonly affected when considering LVO strokes). These include the lateral lenticulostriate, frontal pars of middle cerebral artery, parietal pars of middle cerebral artery, temporal pars of middle cerebral artery, occipital pars of middle cerebral artery and insular pars of middle cerebral artery.^38^

From a brain network perspective, we considered two different atlases. The first template divides the brain into grey matter parcels belonging to different resting state networks. The second atlas includes white matter bundles connecting the same resting state networks. The functional atlas was obtained from fMRI sequences of *n* = 1000 subjects described in Yeo *et al.*,^39^ subdivided into 7 and 17 networks. Both parcellations were used in this study to investigate the impact of the prediction assessed with different network dimensions. The seven-network atlas includes limbic (LMB), default mode network (DMN), frontoparietal network (FPN), DAN, ventral attention network (VAN), sensorimotor network (SMN) and visual network (VIS). The 17 network parcellations include peripheral-VIS, central-VIS, SMN-A, SMN-B, DAN-A, DAN-B, VAN-A, VAN-B, LMB-A, LMB-B, control network A, control network B, control network C, DMN-A, DMN-B, DMN-C and temporoparietal. In addition, we included in this functional parcellation the subcortical structures not considered in the original Yeo's parcellation. Specifically, we included structures of the Harvard-Oxford atlas^40^: amygdala, hippocampus, thalamus, caudate, putamen, nucleus accumbens and pallidum. These structures were grouped as a unique parcellation (basal ganglia network) for both 7- and 17-network templates.

The white matter atlas was obtained from fMRI-guided diffusion tractography imaging (DTI) in *n* = 32 healthy subjects and individuates white matter bundles that connect the regions of 13 functionally defined brain networks: the dorsal DMN, the ventral DMN, the left executive control network, the right executive control network, the anterior salience network, the posterior salience network, the arcuate network, the basal ganglia network, the higher VIS, the language network, the precuneus network, the SMN and the visuospatial network.^41^ These white matter bundles connect Yeo's grey matter networks through anatomical pathways. Given the lesion distribution associated with LVOs in the anterior circulation, the cerebellum was excluded from this analysis.

### Statistical analysis

To estimate the prediction value of the lesion on outcome, we calculated the percentage of overlap for each lesion and each parcel belonging to the vascular, functional and structural atlases. To this aim, two measures were computed. The first metric reflects the spatial distribution of the lesion as the lesion percentage encapsulated in each parcel. The second metric represents a volumetric parameter defined as the percentage of each parcel overlapping with the lesion mask. This latter measure was used jointly with the first to compute an exclusion score to identify the parcels with a low lesion load. Specifically, we excluded parcels with a lesion/parcel percentage overlap lower than 5%. In other words, we just considered overlaps involving at least 5% of the lesion or 5% of the parcel. This procedure was repeated for the vascular, Yeo's (7 and 17 networks) and white matter parcels.

We applied a linear regression approach with a regularized Lasso function. In the Lasso regression, we tested three different models corresponding to the frameworks described above (vascular versus network representation). Specifically, we run the analysis considering as the independent set of variables: (i) lesions in the vascular atlas, (ii) lesions in the grey matter networks (7 and 17 parcellations) and (iii) lesions in the white matter networks. The regularization parameter for the Lasso regression (L1) was assessed through a leave-one-out (LOO) cross-validation procedure between a set of 100 values ranging from 1 × 10^−5^ to 1 × 10^5^. Performance was evaluated based on the negative mean squared error. Prediction was assessed using the *R*^2^ score. We run a LOO analysis to compare the standard deviation of the prediction for the different models (vascular atlas versus functional atlas versus structural atlas). Additionally, we used the same procedure to test a reference model including demographic and clinical variables (age, sex, NIHSS at admission). Finally, we tested the value addition of vascular and network representation to demographic and clinical variables.

The relationship between functional outcome and brain features was investigated at the voxel-wise level. For this analysis, we included lesion topography, as well as structural and functional disconnectivity maps computed for each patient through a non-parametric inference using (*n* = 1000 permutations family-wise error (FWE) corrected at threshold free cluster enhancement (TFCE) with a stringent *P-*value <0.01). Before running the analysis, functional disconnectivity maps were threshold at a value of *r* = 0.2, as in our previous study,^37^ while structural disconnection was considered with a value higher than 0.5, corresponding to a 50% probability that the disconnected tracts are reported in more than 50% of the normative sample.^34^

**Figure 1:**
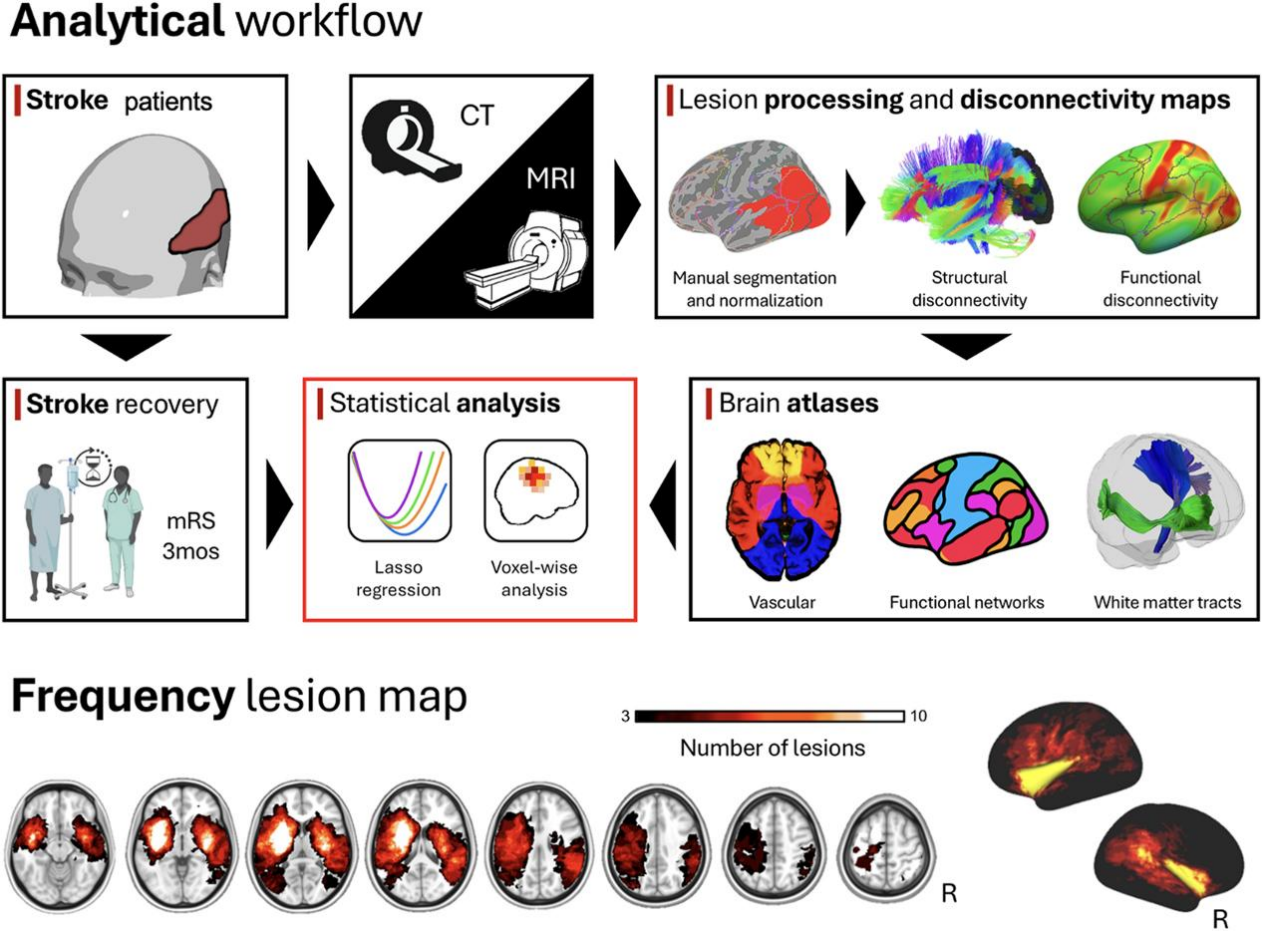
**Analytical workflow of the study and lesion frequency map.** Top panel: Stroke patients underwent CT or MRI scan for lesion identification. Normalized lesion masks were used to compute indirect disconnectivity maps (both structural and functional). Maps were then projected into different brain atlases from vascular, functional and white matter tracts. Patients were followed after 3 months to assess recovery (mRS). Lasso regression and voxel-wise analysis were applied to assess the relationship between lesion-transformed features and disability. Bottom panel: Lesions map in the volumetric (left) and surface (right) space overlaid to a normative template. R, right.

All the procedures were run on Python 3.9 through an ASUS TUF Dash F15 machine [12th Gen Intel(R) Core (TM) i7-12650H 2.30 GHz] running on an Ubuntu 20.04.6 LTS (Focal Fossa) environment. The scikit-learn v1.4.1 and scipy v1.9.1 libraries were utilized within a conda environment. Imaging data were manipulated through the NiBabel library. Figure 1 depicts the analytical workflow.

## Results

### Study sample

A total of *n* = 70 patients were included. The age of the patients was 73 ± 12 years (i.e. mean ± SD) and 53% were females and 47% males. The majority were hypertensive, *n* = 8 (11%) suffered a heart attack, *n* = 25 (36%) had atrial fibrillation and *n* = 14 (20%) were diabetics. The mean pre-stroke mRS pre-event was 0.5 ± 0.9 (i.e. mean ± SD) and the mean NIHSS at presentation was 13.8 ± 6.8 (mean ± SD). The majority (i.e. 60%) presented a known time of onset with a mean time to recanalization of 351.5 ± 60 min (mean ± SD). The remaining patients presented either a wake-up stroke or an unknown time of onset. For this subgroup of patients (*n* = 28), the midpoint time between last-known-well and revascularization was 324.3 ± 93 min (mean ± SD). These slightly overall extended time intervals reflect the exclusion of patients with un-available advanced perfusion imaging (see the Inclusion Criteria) and are in line with Italian^42^ and International^43^ guidelines for acute revascularization diagnostics and treatment. Median ASPECTS was 8 (IQR 6–9). The rate of successful recanalization was 95% as measured by the eTICI scale (i.e. >TICI2b50). After treatment, one-third of the patients developed different types of intracerebral bleeding assessed by the Heidelberg Bleeding Classification, and nearly half presented mild systemic complications. Finally, six patients died before discharge. At discharge, the mean NIHSS score was 5 ± 5.9 (mean ± SD) while the mRS was 2.5 ± 2.1 (mean ± SD). At 3 months, the mean mRS was 1.7 ± 1.6 (mean ± SD), including 56% of patients with poor outcome (i.e. mRS 3–6). These characteristics are comparable with recently published retrospective stroke cohorts who underwent MT.^22^ See Supplementary Table 1 for the complete clinical description of the study population.

### Stroke anatomy

We acquired a total of 40 post-MT CT scans and 30 post-MT MRIs. The mean time of imaging acquisition after the event was 7 ± 3.5 days (mean ± SD). The patients presented ASPECTS of 8.4 ± 1.6 (mean ± SD). Lesions were mainly located in the MCA, deep in the parietal and frontal lobe affecting the central white matter, the basal ganglia and the thalamus, in line with previous studies.^44-47^ Forty patients presented with a left hemisphere stroke, while 30 patients presented lesions in the right hemisphere. Most patients presented an LVO of the MCA (i.e. 44% of M1 and 27% of M2) as detected by CT angiography. See Supplementary Table 2 for the radiological description of the clinical population. Figure 1 reports the frequency map of lesions as well as the analytical workflow of the methodology.

### Atlas-based functional outcome prediction

Lasso regression was computed for lesions mapped onto a (i) vascular atlas, (ii) Yeo's functional networks (7 and 17 templates) and (iii) Figley's structural connectivity networks.

**Figure 2:**
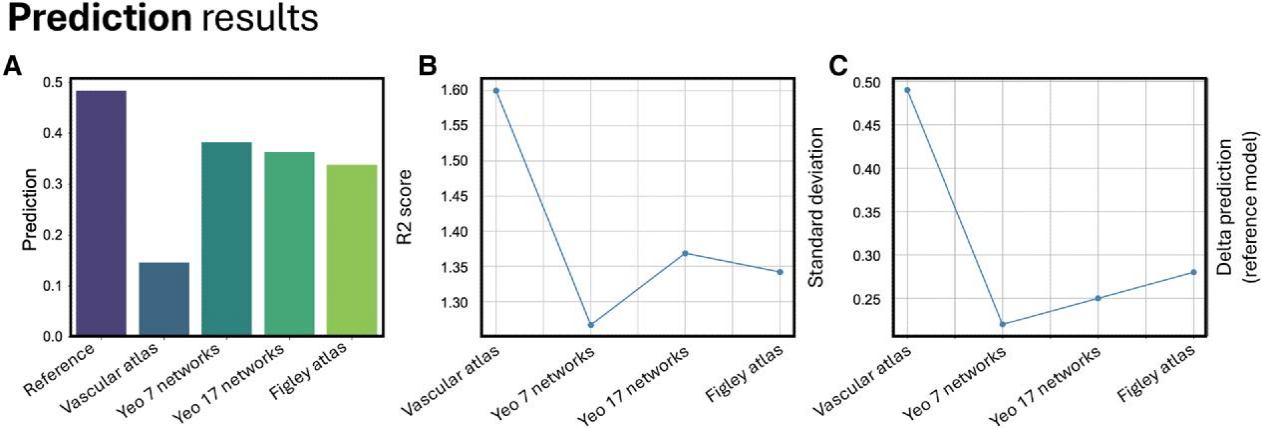
**Predictive performance of lesion-derived models.** (**A**) Prediction scores for the five different models based on the reference model and the atlases considered in the study (single best performance score). (**B**) Standard deviation scores for the LOO prediction scores based on the four models. (**C**) Increase in prediction of the four models after including demographic and severity variables as covariates.

The prediction of the mRS was the most robust for Yeo's functional atlas (*R*^2^ = 0.382), followed by Figley's structural atlas (*R*^2^ = 0.338), while the vascular atlas provided the lowest prediction for functional outcome (*R*^2^ = 0.146) (Fig. 2). The results for the functional template were confirmed using the 17 network parcellation (*R*^2^ = 0.363). Qualitatively, the LOO results showed the lowest standard deviation for the prediction based on Yeo's seven networks, while they were the highest for the vascular atlas (Fig. 2B). The benchmark model including demographic and clinical variables explained the highest variance (*R*^2^ = 0.484). When including these variables in the atlas-based predictions, the vascular atlas showed the largest improvement in accuracy from the baseline (Δ prediction = 0.48), due to the model low performance when relying solely on vascular information. In contrast, the accuracy gains for the network-based models, which already performed better in the baseline condition, were smaller and comparable (Δ prediction around 0.20). These results are presented in Fig. 2. Overall prediction values were similar across all models (i.e. *R*^2^ ≈ 0.6). Interestingly, the admission ASPECTS (range 1–10) of these patients was weakly correlated with the 3-month mRS (*r* = 0.130), explaining a negligible amount of behavioural variance (*R*^2^ = 0.017), in line with the low performance of the vascular atlas.

**Figure 3:**
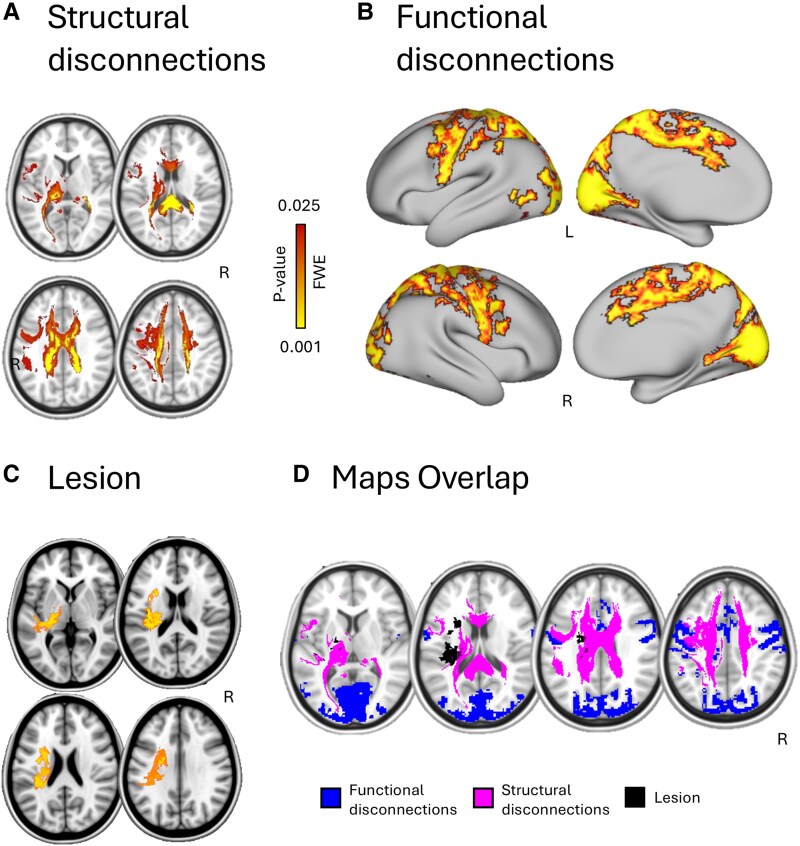
**Anatomical correlates of 3-month disability following stroke.** We describe regions showing significant correlation with disability at 3 months in the stroke sample (linear correlation with *n* = 1000 permutations FWE-corrected at TFCE with a *P-*value of 0.01, *N* = 70). (**A**) Structural connectivity maps of white matter tracts in axial view. (**B**) Functional disconnectivity regions. (**C**) Damaged voxels (lesion). (**D**) Overlap between the three maps (functional: blue; structural: pink; lesion: black) on the MNI template. R, right.

The voxel-wise lesion topography maps identified regions significantly associated with functional outcome at 3 months. These involved bilaterally the corona radiata and left corticospinal tract. No significant voxel survived when considering the negative relationship between lesion topography and mRS (*P* > 0.05) (see Fig. 3C). Functional disconnectivity map showed regions positively associated with the mRS score. Regions of functional disconnection across the entire group localized to the frontal eye fields, the precentral/postcentral gyri, the supplementary motor area, the superior and inferior parietal lobe, the cuneus and precuneus, the calcarine cortex, the inferior occipital lobe, the middle temporal gyrus, the right inferior temporal lobe, the cingulum and the cerebellum. From a network perspective, the mRS significantly correlated with different networks: VIS (*R*^2^ = 0.379; *P* < 0.05), SMN (*R*^2^ = 0.340; *P* < 0.05) and DAN (*R*^2^ = 0.318; *P* < 0.05) (Fig. 3B). The relationship between connectivity and mRS was positive, i.e. stronger functional disconnection higher (poorer outcome) mRS. No significant voxels survived for the opposite relationship (*P* > 0.05).

**Figure 4:**
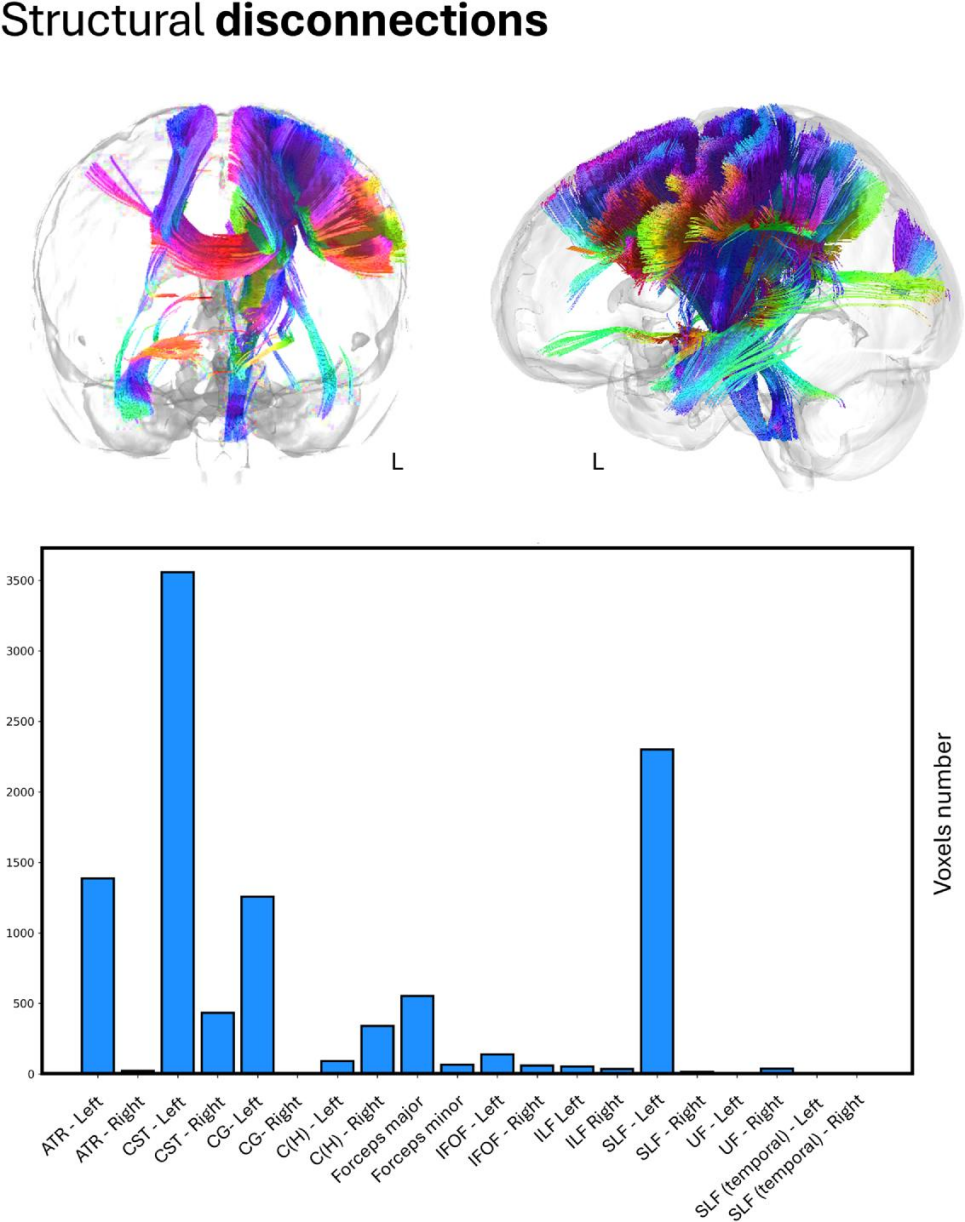
**White matter fibre bundles related to disability.** The top panel shows the coronal and sagittal view of the white matter fibre bundles significantly associated with the mRS (linear correlation with *n* = 1000 permutations FWE-corrected at TFCE with a *P-*value of 0.01, *N* = 70). The bottom panel displays the list of the involved tracts by calculating the binarized overlap with the Johns Hopkins University (JHU) tracts. The disconnected maps considered were thresholded at 50%. ATR, anterior thalamic radiation; CST, corticospinal tract; CG, cingulate gyrus; C(H), cingulum (hippocampus); IFOF, inferior fronto-occipital fasciculus; ILF, inferior longitudinal fasciculus; SLF, superior longitudinal fasciculus; UF, uncinate fasciculus.

The voxel-wise structural disconnectivity analysis extended the, previously described, lesion topography results, showing that several white matter bundles significantly correlated with the 3-month mRS (Figs. 3 and 4). These include the corticospinal tract, corpus callosum, corona radiata, thalamic radiation and left inferior and superior longitudinal fasciculus (Fig. 4). Higher levels of structural disconnection corresponded to higher mRS and disability. No significant voxel survived when considering the negative relationship between structural disconnection and mRS (*P* > 0.05).

## Discussion

This exploratory study compared the predictive ability of lesion location on functional outcome and disability in three different brain spaces: a vascular (ASPECTS-like) atlas, a functional grey matter atlas and a structural white matter atlas. In addition, we described the anatomy of lesion topography and structural and functional disconnection patterns related to long-term functional outcome in stroke patients with LVO of the anterior circulation who underwent MT.

### Vascular versus large-scale network topography prediction on stroke outcome

Lesion prediction on the mRS at 3 months was most robust for Yeo’s functional atlas (*R*^2^ = 0.382 and 0.363, respectively, for the 7- and 17--network parcels). It was intermediate for the structural white matter atlas (*R*^2^ = 0.338). The vascular model provided the worst prediction on outcome (*R*^2^ = 0.146). Yeo’s functional atlas provided the closest prediction to the benchmark model including demographic and clinical variables. Interestingly, the vascular framework had the lowest prognostic value even though the dimensionality of the parcels used was higher (*n* = 32) as compared to the number of networks (*n* = 7 and 17) or structural connectivity systems (*n* = 13). Our results suggest that damage to specific vascular territories is not the critical variable for prognosis, while the structural/functional organization of the brain metabolically supported by vascular supply may provide a better explanation for recovery. This is in line with previous studies that have shown that behavioural symptoms following stroke do not fit classic vascular syndromes.^48^ Rather, behavioural deficits across patients correlate across latent variables that account for a high percentage of behavioural variability.^47^ This low dimensionality of behavioural deficits is partially explained by common phenotypes of brain functional alterations that relate to a large-scale brain network organizational framework and include the weakening of inter-hemispheric functional connectivity and the decrease of intra-hemispheric segregation.^27,49^ In addition, recent work on post-stroke rehabilitation has emphasized the role of structural reserve on the neuroplastic potential of these patients.^30,31^ This concept describes the extent to which neural pathways and relays spared by the lesion contribute to recovery in an individual patient. These regions could contribute following an inter-hemispheric imbalance or vicariation model of recovery depending on the amount of spared functionally related regions. In this context, our results provide critical evidence in this direction as our model stratifies recovery of patients according to the percentage of spared resting state networks. This evidence prompts a change of perspective also in the acute phase of stroke, going beyond the measure of variability in severity of damage from both a volumetric and topographical point of view.^17,19,28^ This means that we rather focus on measures of residual capacity of neuroplastic potential when assessing direct anatomical damage and connectome disruption in acute stroke. This is a particularly promising prospect for future experimental interventions (i.e. non-invasive brain stimulation), commonly considered as plasticity-modifying approaches,^50-52^ involving structurally intact but functionally relevant regions of the brain.

### Structural and functional disconnection patterns related to post-stroke outcome

Significant patterns of functional disconnection related to the mRS mainly involved the sensorimotor system, the DAN and the VIS. In particular, the mRS significantly correlated with the visual (*R* = 0.379, *P* < 0.05), sensorimotor (*R* = 0.340, *P* < 0.05) and dorsal attention (*R* = 0.318, *P* < 0.05) networks. The voxel-wise lesion damage analysis confirmed the well-described involvement of the corticospinal tract and corona radiata.^22,53^ However, the white matter disconnection analysis extended these results, additionally identifying significant associations with the anterior callosal fibres, thalamocortical pathways, uncinate fasciculus, forceps major and bilateral inferior/superior longitudinal fasciculus. These findings substantially explain the described functional abnormalities involving the DAN and VIS networks through the disconnection of long association tracts. The overlap of the described neural correlates underscores their close anatomical relationship (see Fig. 3D). While a large body of literature has focused on explaining different behavioural deficits after damage to specific white matter tracts or cortical areas,^54,55^ most of the research has mainly concentrated on the pivotal role of the corticospinal tract for the prediction on clinical scales of outcome used in most RCTs (i.e. the mRS).^22,56,57^ Our results extend these findings significantly as disability evidently loads on semantic, visuomotor and visuospatial functions and the subserving anatomical correlates. Specifically, the involvement of part of the left anterior thalamic radiation, uncinate fasciculus and inferior longitudinal fasciculus bilaterally is consistent with language deficits that may impact long-term outcome and disability. The bilateral disconnection of the superior longitudinal fasciculus and forceps major instead is in line with spatial attention deficits affecting chronic disability.^58^ Future work should consider the role of additional eloquent tracts and cortical regions on outcome, as described here. Interestingly, we identified regions across multiple vascular territories well beyond our anatomical distribution. The described RSNs and white matter bundles include multiple vascular territories (i.e. the anterior cerebral artery and posterior cerebral artery). In addition, it is worth noting that we found a significant association of functional and structural disconnection with a functional outcome evaluation that globally measures disability and has been frequently criticized for being geared towards motor functions (i.e. the mRS).^9^ We hypothesize that the application of clinical tools extending beyond the physical sequelae of stroke (i.e. the Stroke Impact Scale, the Geriatric Depression Scale, the Oxford Cognitive Screen)^59-62^ could provide additional insights on significant neural correlates for recovery.

### Limitations and future directions

Our exploratory study has several limitations worth noting. The retrospective study sample can be considered only small to medium in the stroke literature. Future work in prospective samples is needed to assess the reproducibility of these results. MRI should be preferred to CT scans (i.e. 57% of scans were CTs in this work) to increase the accuracy of the results. Further, while FLAIR images provide valuable lesion delineation, especially in an early sub-acute setting, diffusion-weighted imaging may have yielded slightly different results, highlighting the need for future studies to assess the impact of subtle differences in lesion delineation. Similarly, the use of an arbitrary threshold for structural disconnections should be carefully evaluated, although previous studies have reported comparable results using different thresholds.^63-65^ In addition, it is important to acknowledge that the structural disconnection analysis was based on preselected tracts transformed to a volumetric voxel-probability space, which may have failed to capture the full variability of white matter anatomy.^66^ Damage to different vascular territories (i.e. cerebellum and posterior circulation strokes) could be assessed by future studies considering a similar framework to provide a more comprehensive description of the topographical correlates related to the functional outcome. One limitation of the Lasso approach is its tendency to retain only one variable among highly correlated predictors, which may lead to the arbitrary exclusion of informative features; this should be considered by future studies when interpreting the importance of individual brain parcels. Finally, in our sample the ASPECTS was negligibly associated with the 3-month mRS (*r* = 0.130, *R*^2^ = 0.017). We argue on the potential of a future translation of our network-based framework in a pre-treatment setting (i.e. for MT treatment eligibility and prognosis). In this context, vascular-based assessments like the commonly used ASPECTS and perfusion-related volumetric features are likely to be less sensitive to behaviour and recovery. This hypothesis would require a prospective study in which eligibility for treatment should be assessed on either ASPECTS or a network-based atlas. While promising, it must be noted that our results derive from the comparison with an ASPECTS-like atlas^38^ in the acute phase of stroke. The ASPECTS is a semi-quantitative measure that can detect early alterations of irreversible damage, following the topographical distribution of the most susceptible areas for ischaemia. This may not be equally true for a network-based atlas that does not follow a vascular distribution. In this context, the future application of large-scale network measures on hyper-acute imaging (i.e. CTP core and penumbra areas) will provide stronger evidence for an effective clinical translation.

## Conclusion

The introduction of MT in stroke treatment has provided clinicians with a robust therapeutic resource.^50^ However, approximately half of the patients who undergo this procedure have poor long-term clinical outcomes. The description of valuable predictive factors has modified the pool of eligible patients, improved treatment effects and provided useful prognostic information. In addition, over the last few years, large-scale network disruption measures have shown consistent post-stroke predictive validity on many behavioural deficits. In line with this work, we investigated the predictive ability of lesion location on functional outcome in different brain spaces: a vascular atlas, a functional grey matter atlas and a structural white matter atlas. In addition, we describe lesion topography and structural and functional disconnection patterns related to a clinically applicable functional outcome measure (i.e. the mRS at 3 months). We observed that topographical data provided a robust outcome prediction only when considered in a functional network framework (i.e. grey matter and white matter atlas). These exploratory results provide significant clinical and theoretical implications. They describe the role of eloquent white matter tracts and cortical regions on outcome, beyond solely the motor domain of function and the underlying corticospinal tract. Most importantly, they suggest a change of perspective in the acute phase of stroke, complementing measures of damage after stroke by focusing on the evaluation of the residual capacity for neuroplastic potential as a fundamental source of variability in recovery.

## Supplementary Material

fcaf285_Supplementary_Data

## Data Availability

All data used and reported in the present study are available from the authors upon reasonable request. Codes generated and used in this work are available in this public repository: https://github.com/pinilorenzo.
