# Supplementary material for: Large-scale network topography of stroke predicts functional outcome after mechanical thrombectomy
Source: Brain Commun. 2025 Aug 28;7(4):fcaf285. doi: 10.1093/braincomms/fcaf285 (PMC12391757; doi:10.1093/braincomms/fcaf285)
Supplement: fcaf285_Supplementary_Data [file fcaf285_supplementary_data.pdf]

SUPPLEMENTARY MATERIAL

Supplementary Table 1. Patient clinical and demographic characteristics

**Clinical and demographic characteristics**	
Age (+-SD), y	73 (12)
Sex: male, no. (%)	33 (47)
NIHSS at admission (mean +- SD)	13.8 (6.8)
Pre-mRS (mean +- SD)	0.5 (0.9)
Time to recanalization with known onset (mean +- SD)	351.5 (60.2)
Time to recanalization with unknown onset or wake-up stroke (mean +- SD)	324.3 (93)
NIHSS at discharge (mean +- SD)	5 (5.9)
mRS at discharge (mean +- SD)	2.5 (2.1)
mRS at 3 months (mean +- SD)	1.7 (1.6)
Risk factors	
Arterial hypertension (%)	44 (63)
Diabetes (%)	14 (20)
Atrial fibrillation (%)	25 (36)
History of ischemic myocardial infarction (%)	8 (11)
Hypercholesterolemia (%)	25 (35)

Supplementary Table 2. Radiological description of the clinical population

**Radiological characteristics**	
ASPECTS (mean and SD)	8.4±1,6
Lesioned hemisphere; left (%)	40 (57)
Vessel occlusion on CTA; n(%)	
• M1 occlusion<br>• M2 occlusion<br>• A1 occlusion<br>• A2 occlusion<br>• ICA occlusion<br>• Intra-extra (tandem) occlusion	30(44)<br>19 (27)<br>1(1)<br>1(1)<br>3 (4)<br>16 (23)
Successful recanalization (>= TICI score 2b) (%)	67 (95)
